# Cutaneous lupus erythematosus following immunoglobulin therapy in dermatomyositis

**DOI:** 10.1111/1346-8138.17553

**Published:** 2024-11-25

**Authors:** Eaman Alhassan, Stratos Christianakis, Brittney DeClerck, Ashley B. Crew

**Affiliations:** ^1^ Division of Rheumatology, Department of Medicine University of Southern California Keck School of Medicine Los Angeles California USA; ^2^ Division of Rheumatology and Clinical Immunology, Department of Medicine University of Pittsburgh Medical Center Pittsburgh Pennsylvania USA; ^3^ Department of Dermatology University of Southern California Keck School of Medicine Los Angeles California USA

**Keywords:** cutaneous, dermatomyositis, immunoglobulin, lupus, myositis

A 57‐year‐old woman with a remote history of follicular lymphoma in remission, treated with rituximab with subsequent diagnosis of hypogammaglobulinemia, presented to the Rheumatology‐Dermatology clinic for evaluation of an autoimmune disease. The patient reported experiencing rash, myalgias, and proximal muscle weakness for 3 months. Physical examination was remarkable for poikiloderma over the neck and chest (V‐sign), upper back (shawl sign), upper arms (sleeve sign), and lateral thighs (Holster sign). She had normal muscle strength. Laboratory work‐up demonstrated normal complete blood count, comprehensive metabolic panel, creatine kinase, and aldolase levels. A myositis‐specific antibody panel and antinuclear antibody tests were negative. The patient was diagnosed with amyopathic dermatomyositis. Because of her lymphoma history and skin‐predominant presentation, intravenous immunoglobulin (Ig) therapy was administered. Three days after receiving the Ig therapy, the patient developed distinct, well‐defined, violaceous, juicy appearing plaques on her face, chest, abdomen (Figure 1a), back (Figure 1b), and arms. Ig therapy was held. Skin biopsy of the new lesions showed interface dermatitis (Figure 1c) with increased dermal mucin and karyorrhectic debris consistent with discoid lupus erythematosus. Oral and topical corticosteroids were added with improvement in her skin lesions (Figure 1d).

**FIGURE 1:**
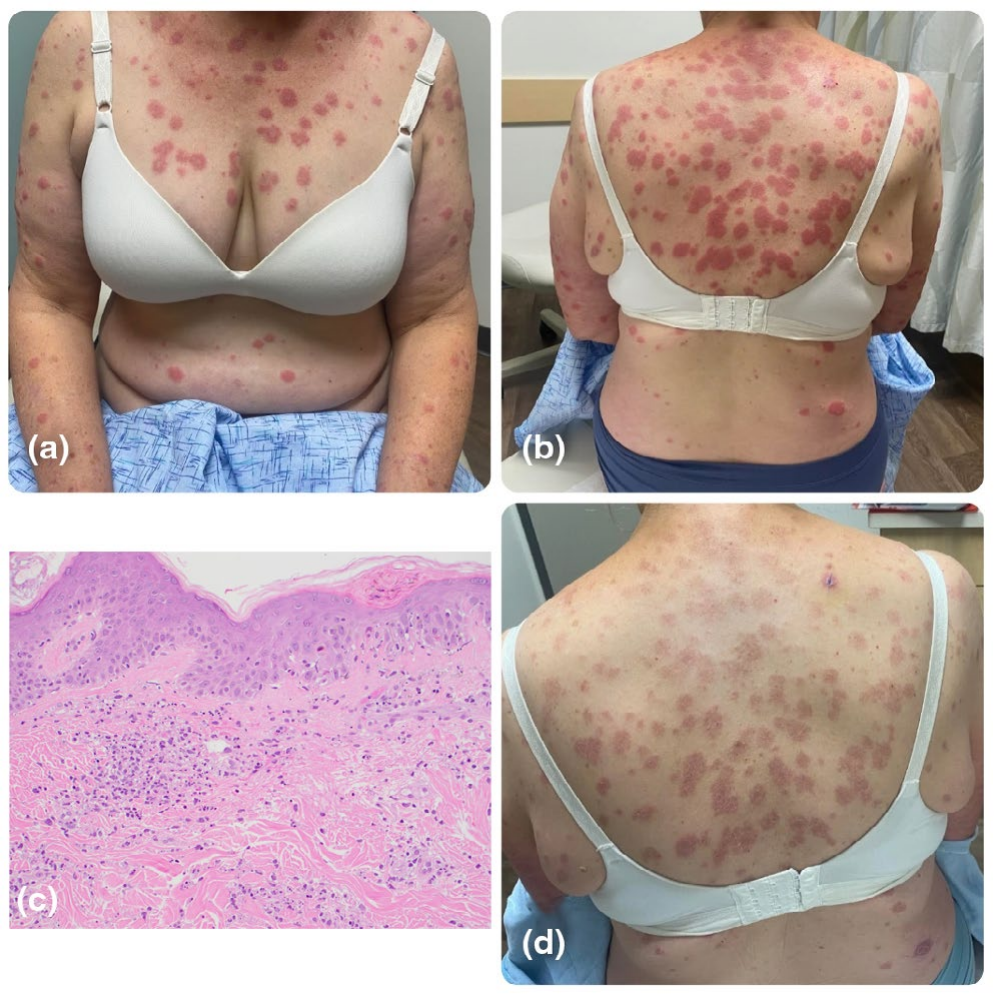
A. Well‐defined, violaceous plaques on the chest and abdomen. B. Well‐defined violaceous plaques on the back. C. Skin biopsy from the upper back lesion showing interface dermatitis (magnification value is 20x). D. Skin lesions after holding Ig therapy and starting oral and topical corticosteroids.

A causative link between Ig G treatment and cutaneous lupus erythematosus has been reported in patients receiving Ig treatment for chronic inflammatory demyelinating polyneuropathy.^1^, ^2^ The rash has been reported to improve after discontinuing Ig treatment or switching to another Ig brand. Van der Molen et al. reported the possible presence of Sjögren's‐syndrome‐related antigen (SSA) antibodies in Ig preparations.^3^ While the mechanism of induction of cutaneous lupus erythematosus in patients being treated with Ig therapy is not well understood, one hypothesis is that the presence of SSA antibodies in Ig therapy may induce cutaneous lupus in a manner similar to cutaneous changes in neonatal lupus.

## CONFLICT OF INTEREST STATEMENT

The authors declare no conflict of interest related to this work.
